# The Weaker Sex? The Propensity for Male-Biased Piglet Mortality

**DOI:** 10.1371/journal.pone.0030318

**Published:** 2012-01-17

**Authors:** Emma M. Baxter, Susan Jarvis, Javier Palarea-Albaladejo, Sandra A. Edwards

**Affiliations:** 1 Animal Behaviour and Welfare, Scottish Agricultural College, Edinburgh, United Kingdom; 2 Royal (Dick) School of Veterinary Studies, University of Edinburgh, Easter Bush, Midlothian, United Kingdom; 3 Biomathematics and Statistics Scotland, Edinburgh, United Kingdom; 4 School of Agriculture, Food and Rural Development, Newcastle University, Newcastle upon Tyne, United Kingdom; University of Turku, Finland

## Abstract

For the most part solutions to farm animal welfare issues, such as piglet mortality, are likely to lie within the scientific disciplines of environmental design and genetic selection, however understanding the ecological basis of some of the complex dynamics observed between parent and offspring could make a valuable contribution. One interesting, and often discussed, aspect of mortality is the propensity for it to be sex-biased. This study investigated whether known physiological and behavioural indicators of piglet survival differed between the sexes and whether life history strategies (often reported in wild or feral populations) relating to parental investment were being displayed in a domestic population of pigs. Sex ratio (proportion of males (males/males+females)) at birth was 0.54 and sex allocation (maternal investment measured as piglet birth weight/litter weight) was statistically significantly male-biased at 0.55 (t_35_ = 2.51 P = 0.017), suggesting that sows invested more in sons than daughters during gestation. Despite this investment in birth weight, a known survival indicator, total pre-weaning male mortality was statistically significantly higher than female mortality (12% vs. 7% respectively z = 2.06 P = 0.040). Males tended to suffer from crushing by the sow more than females and statistically significantly more males died from disease-related causes. Although males were born on average heavier, with higher body mass index and ponderal index, these differences were not sustained. In addition male piglets showed impaired thermoregulation compared to females. These results suggest male-biased mortality exists despite greater initial maternal investment, and therefore reflects the greater susceptibility of this sex to causal mortality factors. Life history strategies are being displayed by a domestic population of pigs with sows in this study displaying a form of parental optimism by allocating greater resources at birth to males and providing an over-supply of this more vulnerable sex in expectation of sex-biased mortality.

## Introduction

Applying ecological theories to situations concerning domestic livestock is a method of addressing applied biological issues in farm animal welfare [1], [2]. Piglet mortality is one such issue still to be effectively addressed and, although solutions are likely to lie within the scientific disciplines of environmental design and genetic selection, understanding the ecological basis of some of the complex dynamics observed between parent and offspring could make a valuable contribution. One interesting, and often discussed, aspect of mortality is the propensity for it to be sex-biased.

Life history theories predict that there will be sex-biased mortality as a result of the differential costs and benefits of raising the two sexes. The two main and applicable theories are: (i) an adaptive manipulation of the sex ratio (% of males) by mothers unable to rear successful sons [3] and; (ii) differential energetic requirements between the sexes in a sexually dimorphic species, where the larger sex are more susceptible to food shortages associated with their faster growth rates and increased nutritional requirements [4]. The first of these theories, the Trivers-Willard Model (TWM) is frequently cited, and is based on the premise that reproductive success is realised in the sex with the greater reproductive returns. It states that, in polygynous species, female offspring in relatively poor condition are expected to realise greater reproductive success than males in a similar condition. Thus, assuming that parent and offspring condition are interrelated and that circumstances in early life impact upon later reproductive success, poor-quality parents should preferentially invest in daughters. This model has been documented frequently and numerous reviews cite examples from a range of taxa [5]–[7]. However, assumptions based on the TWM become problematic in species producing litters or broods, particularly in the pig (Sus scrofa), where litters are often large [8], [9]. Moreover, the opportunity for sex-specific maternal intervention during the postnatal period from birth to weaning is limited in the pig, and would involve either specific allocation of resources or specific mortality. Sows are not able to individually recognise piglets in their litter for the first seven days of life but identify the nest site [10] and care for piglets within it. Individual bonds do not develop, but a general olfactory recognition of the litter exists [11]. Therefore it seems unlikely that discrimination would exist at this postnatal stage and general litter size adjustment may be more important than sex ratio variation for ensuring reproductive fitness [9]. Prenatal mortality, often discussed in terms of birth sex ratios and sex allocation (the resources invested in offspring), may support the TWM, as there are greater opportunities for sex-specific maternal investment. This is a topic of considerable discussion [12]–[18].

The second theory of males being more susceptible to mortality because of higher energetic demands associated with sexual dimorphism [4] would appear a more parsimonious explanation when observing polytocous species like the pig, at least with respect to postnatal sex-biased mortality. Darwin [19] stated that “sexual selection depends on the success of certain individuals over others of the same sex”. The development of secondary sexual characteristics that enhance the individual's chance of reproductive success, are evident in polygynous species, such as the pig. Secondary sexual characteristics often cited in the literature include the exaggerated plumage of male birds of paradise or the excessive armoury of male red deer; the former used passively to attract mates, the latter used aggressively to compete with other males for mates [20]. In the pig, large body size and tusks are the secondary sexual characteristics requiring investment and only individuals with the strongest secondary sexual characteristics will be successful in competing for mates and subsequently contributing their genes to a population. Thus, the larger sex in a sexually dimorphic species, will be subject to greater selection pressure in order to realise greater reproductive fitness. The investments in secondary sexual characteristics that determine reproductive fitness have important ramifications for patterns of growth, development, mortality and sex ratios [21].

Fernández-Llario et al. [22] warned against functional studies on domestic animals, as the artificial environment could realise non-adaptive patterns rather than ecological strategies. However, in livestock kept extensively and exposed to a more varied environment, it is possible that strategies evolved by their wild counterparts are functionally exhibited. The behavioural repertoire of domestic pigs has changed very little through domestication [23], [24], although the thresholds of expression have been altered [25]. Pigs can alter their behavioural decisions based on environmental cues [26]–[28], and this could also be the case with ecological strategies that might prove adaptive in certain environments. Though globally the predominant farrowing and lactation systems are indoor, intensively managed farrowing crates, the commercial outdoor environment for domestic pigs offers an opportunity to study modern genotypes under more natural settings to investigate whether life history strategies continue to be displayed.

Given the theories already discussed regarding sex-biased mortality, it is therefore relevant to determine whether male and female piglets differ in the extent to which they exhibit survival indicators known to be critical to the neonate. To ensure postnatal survival, the piglet must negotiate a challenging extrauterine environment, which includes a 15–20°C drop in temperature, the movements and behaviour of an unpredictable mother [29], [30] and competition for limited food resources (high quality teats) with potentially numerous siblings [31]. A piglet that is slower to reach the udder, slower to take in colostrum, unable to respond to maternal cues and ineffective in response to sibling rivalry will compromise its survival. Preserving homeothermy [32] and displaying high vigour [33]–[35] are essential adaptations to extrauterine life and ultimately survival. Thus, this study investigated the behavioural and physiological characteristics of piglet survival, in a commercial, outdoor farrowing system, to determine whether there were differences between the sexes. It aimed to investigate whether domestic populations of pigs still express ecological strategies for reproductive success, similar to their wild counterparts by: i) examining if a sex ratio is present at birth, ii) examining if sex allocation bias occurs and iii) examining whether sex-biased mortality occurs.

## Materials and Methods

### Ethics Statement

This study was reviewed and approved by SAC's Ethical Review Committee (approval ID: ED AE 5/2005).

### Study Subjects

The experiment took place on an outdoor pig farm in Aberdeenshire, with sows and piglets managed under typical commercial conditions. A total of 478 progeny from 36 parturitions (known as and hereafter referred to as farrowings) of fifth parity, Landrace×Large White×Duroc sows, were subject to detailed study over a period of four months. All sows were artificially inseminated in a nearby indoor facility. After being inseminated, sows were grouped together (approximately 20 sows per group) in outdoor gestation paddocks. Sows were moved from gestation paddocks to individual farrowing paddocks approximately 10 days before the farrowing due date. The individual farrowing paddocks were approximately 20 m×20 m, separated by electric fences. Farrowing occurred in double-skinned insulated huts, with sloped wooden walls and a floor area of 3.09 m^2^ (a full description and diagram of the housing conditions and behavioural recording protocols is available in [36]). Huts were initially bedded with high quality barley straw to a depth of approximately 10–12 cm. This was replenished when needed. Once a day, pregnant sows were offered three kg of a diet containing 12.74% CP, 13.32 MJ DE kg^−1^. After farrowing, lactation diet (17% CP, 13.75 MJ DE kg^−1^) was offered at a rate of three kg per day followed by 0.5 kg increments each day until seven kg, and then followed by one kg increments each day up to a maximum of 12 kg until weaning. Sows had ad libitum access to water from a trough. Farrowing was allowed to occur naturally, although staff were present for data collection. Approximately 24 h after piglets were born they underwent scheduled husbandry procedures, including teeth clipping, tail-docking and ear-tagging with an identification number. Cross-fostering according to functional teat number was a husbandry method that could be used if necessary, however no fostering was done during the course of this study as litter size never exceeded functional teat number. Piglets were weaned at no less than 26 days old.

### Behavioural and Physiological Data Collection

Back-fat measurements (taken ultrasonically at the P_2_ position; 6.5 cm from the midline at the level of the last rib) and condition scores (1–5 scale of increasing condition; [37]) were recorded on the sows prior to entry into farrowing accommodation and at weaning. Before the sows were moved into farrowing paddocks, the huts were modified for safe data collection and 24 h filming. Cameras had infra-red facilities which allowed 24 h filming without disturbing the sow and her piglets (further details are given in [36]). Video recording started approximately three days before the farrowing due date and continued for two days post-farrowing. Behavioural and physiological data were collected from the piglets during the farrowing period and for 24 h post-farrowing. Piglets were monitored until weaning. Piglets dying between birth and weaning were recorded and cause of death was ascertained by post-mortem analysis. All farrowings were attended and therefore stillborn piglets could be recorded accurately. If confirmation of stillbirth was needed, post-mortem examination of the lungs was carried out. Mummified piglets were recorded but not included in any analyses. Physiological data collection began at the birth of the first piglet for each litter.

Information collected within litter consisted of litter size (LS), birth order (BO), birth interval (BI) (the interval between each piglet being born) and cumulative farrowing duration (CFD) (the elapsed time between the birth of the first piglet and each subsequent piglet). Immediately after birth, the vitality (V) of the piglet was visually assessed and scored using a categorical scale:

1 =  No movement, no breathing after 15 seconds;
2 =  No body or leg movement after 15 seconds, piglet is breathing or attempting to breathe (coughing, spluttering, clearing its lungs);
3 =  Piglet shows some movement, breathing or attempting to breathe and rights itself onto its sternum within 15 seconds;
4 =  Good movement, good breathing, piglet attempts to stand within 15 seconds.

A blood glucose sample was taken at birth (BG) from the umbilical cord as a mixture of venous and arterial blood and was analysed using a blood glucose monitor (Accu-chek Advantage Glucose Meter™, Roche Diagnostics, West Sussex, UK). Piglets were weighed at birth (BW) and a crown to rump length (CRL) was measured (the supine length of the piglet from the crown of its head to the base of its tail). From these measurements, ponderal index (PI; birth weight (kg)/crown-rump length (m)^3^) and body mass index (BMI; birth weight/crown-rump length^2^) were calculated for each piglet. Piglet gender was noted and birth rectal temperature was recorded (BT) using a digital thermometer (BF-169 Flexible tip digital thermometer, Farlin Infant Products Corporation, Taiwan). The piglet then underwent a righting response test (R): the piglet was placed gently on its back onto a concave beanbag, then it was released and its latency to right itself onto its feet was timed. The piglets were given 15 seconds to right themselves, if they failed to do this within 15 seconds a latency of 16 seconds was noted. Once a piglet had undergone these measurements, a birth order number was written on its back and it was returned to the sow at the birth site. Processing each piglet in this way took approximately four minutes. Piglets were processed with care by trained staff to minimise handling stress and any confounding effects in the data.

Piglet behaviour data were collected from video play-back and latencies to key landmark behaviours were recorded from birth, with processing time subtracted from these latencies:

- Latency to reach the udder (U): time between birth and when contact between the piglet's snout and the sow's udder was made.
- Latency to reach a functional teat (T): time between birth and when firm contact between the piglet's open mouth and a functional teat (not a dummy or inverted teat) was made.
- Latency to suckle (S): time between birth and firm and prolonged (over 2 seconds) contact between the piglet's mouth and a functional teat was made. The piglet typically adopted a brace position, rapid mouth and muzzle motions occurred and frantic teat-seeking stopped.

Rectal temperatures were recorded at 1 h (T1) and 24 h (T24) after birth. Approximately 24 h after birth, a second blood glucose (G24) sample was taken when the piglet was tail docked; after the tip of the tail was docked this discarded tissue was used to provide a blood sample. Piglets were weighed again at 24 h old (W24) and the percentage weight change over this 24 h period was calculated. The piglets were then weighed again at four days old (W4) and at approximately 28 days old when being weaned (WW).

### Statistical Analyses

Sex ratios (males/males+females) were analysed using the test of equal proportions based on the Pearson's Chi-square statistic with continuity correction. The same test was used to simultaneously compare within-litter sex ratios to 1∶1 equality. Note that some litters did not reach an adequate size for the test and were ruled out. Sex allocation (weight of males/weight of litter) was analysed by the t-test after checking for normality. Generalized Linear Mixed Models (GLMMs) were fitted by Laplace approximation for models of non-normal data, with appropriate link functions and error structures depending on the nature of the response variable. Continuous responses were fitted by REML with a normal error structure and an identity link function. In all mixed models litter effects were included as a random factor. Statistically significant terms were determined at the usual level of 0.05. Univariate P-values for the fixed effects were determined by asymptotic normal approximation. All statistical analyses were conducted using R version 2.13.1 (R Development Core Team, 2011).

Mortality variables (dead or alive piglets) for each sex were modelled using GLMMs with logit link function and binomial error structure. Analyses were split according to the two distinct mortality types: firstly, numbers of stillborn piglets relative to numbers of surviving piglets (i.e. those that were weaned) were compared. Secondly, numbers of live-born piglets that subsequently died before weaning relative to numbers of surviving piglets were modelled. With the aim of determining survival indicators for each mortality type, candidate variables were grouped into separate blocks (piglet traits, farrowing traits and sow traits; including behavioural traits in the postnatal mortality type). In order to control for potentially confounding sources of variation, GLMMs were fitted on all the variables within a block, also including sex and all two-way interactions. Collinearity diagnostics were carried out based on Spearman correlation and variance inflation index. From these, it was found necessary for BMI to be used as representative of BW, PI and W24 in the piglet traits block; and U and S represented T in the piglet behavioural traits block. The minimal adequate model for each block was sought by model selection based on AIC and likelihood ratio tests.

Mixed models were also used to analyse sex differences between significant survival indicators. For ordinal responses such as BO and CS a cumulative logit link function was specified. BI was fitted to a Poisson error structure and a log link function. Overdispersion was accounted for by adding an observation-level random effect. The simpler Generalised Linear Model (GLM) was used to model responses only varying at litter level.

Weight and thermoregulation over time were modelled by Linear Mixed Models (LMMs) considering sex, weaning survival status, time and their interactions as fixed effects. As random effects we specified a nested structure consisting of litters and piglets within litters allowing for correlation over time. In the thermoregulation case, weight evolution was also included in the model as a covariate by linking BW to both BT and T1, and W24 to T24. To test the relationship between maternal condition (as measured by CS and P_2_ pre-farrowing) and sex ratios at birth Spearman's rank correlations were used.

## Results

### Sex ratios and sex allocation

Of the 478 piglets born, 256 were male and 222 were female and the overall population sex ratio (male/male+female) was 0.54. In a Chi-square test this ratio was not statistically significantly different from 0.5 (χ_1_^2^ = 2.28, P = 0.13). Within-litter sex ratio biases were not statistically significantly different (χ_29_^2^ = 23.06, P = 0.77). Males were observed to be born heavier on average than females (1516 g (±SE 24.8) vs. 1468 g (±SE 23.6), t_438_ = 1.95, P = 0.052) and had statistically significantly higher mean body mass index (20.3 (±SE 0.26) vs. 19.6 (±SE 0.3), t_424_ = 2.66, P = 0.008) and ponderal index (75.6 (±SE 1.2) vs. 72.5 (±SE 1.1), t_424_ = 2.11, P = 0.035). There was a male-biased maternal allocation at birth for mean weight ratio (0.55 (±0.01), t_35_ = 2.51, P = 0.017).

### Mortality

Total preweaning piglet mortality (stillbirths plus subsequent liveborn mortality) was 18.8%, similar to commercial figures reported at the time of the study (16–20% [38]). Within this slightly male-biased piglet sample, more male piglets died on average than females (total of 57 vs. 33, z = 2.06, P = 0.040). This effect arose mainly as a result of postnatal mortality (piglets being born alive but subsequently dying). The reason for death was determined by post-mortem analysis and Table 1 shows the difference between male and female piglets in causes of death, and the significance of sex as a factor in different causes of mortality. There was no evidence for a sex effect on prenatal mortality (stillbirth). It was observed that males tended to get crushed more often by the sow than females and male piglets died more frequently than females from “other” causes. This mortality group was predominated by disease-related conditions (e.g. diarrhoea). By weaning, there was no significant difference in the number of males and females surviving in the population (weaned sex ratio 0.51, χ_1_^2^ = 0.21, P = 0.65). The within-litter weaned sex ratio was also not significantly different to 0.5 (χ_26_^2^ = 21.98, P = 0.69).

**Table 1:** Percentage of total number of piglet deaths classified by the different causes for each sex.

	Females	Males	z-statistic^1^	P-value
**Stillborn**	16.67	12.22	−0.97	0.334
**Crushed**	13.33	27.78	1.68	0.092
**Low viability or starved**	3.33	6.67	0.57	0.569
**Other** 2	3.33	16.67	2.21	0.027

GLMM results indicate the statistical significance of the mean differences between sexes.

1 Statistical test indicates the significance of sex as a determinant of mortality from specific causes of death.

2 Other deaths primarily related to disease.

### Survival Indicators

Surviving piglets were compared with stillborn piglets (Table 2) and with those that died in the postnatal period (Table 3) to determine whether survival indicators differed between the mortality and survival groups. The most important survival indicators varied with mortality type (the test statistics and associated P-values shown for statistically significant survival indicators result from the GLMM minimal adequate model for each block of traits. For statistically non-significant survival indicators, those statistics show the values at which they were removed from the model – Tables 2 and 3). Following collinearity diagnostics the minimal adequate model for traits associated with the piglet, farrowing and the sow were sought; piglet body conformation, specifically body mass index and crown-rump length, and farrowing kinetics (birth order and birth interval) were statistically significant in explaining the variation between stillborn and surviving piglets (Table 2). The final postnatal group model for piglet traits showed that body mass index and rectal temperature at one hour old were the most statistically relevant piglet traits (Table 3), with sex also a statistically significant factor (z = −2.15, P = 0.031). Piglet behavioural traits were not found to have statistically significant effects in the group model but a sex effect was apparent (z = −3.11, P = 0.002). Litter size was a statistically significant farrowing related survival indicator (Table 3), as well as sex (z = −3.18, P = 0.002). Sow traits were not found to have statistically significant effects in the group model but a sex effect was apparent (z = −3.26, P = 0.001). Overall, sex effects were statistically significant with respect to postnatal survival regardless of which block was considered, thus confirming the importance of gender as a factor in postnatal survival.

**Table 2:** Prenatal survival indicators (means and standard errors (SE)) comparing surviving piglets with stillborn piglets.

Covariates	Surviving (±SE)	n	Stillborn (±SE)	n	z-statistic^2^	P-value
Piglet traits						
Birth weight (g)	1552.3 (±17.0)	386	1234.2 (±88.8)	26	-	-
Crown-rump length (cm)	27.5 (±0.1)	374	28.8 (±0.8)	26	2.57	0.010
Body mass index	20.6 (±0.2)	374	14.4 (±0.8)	26	−3.23	0.001
Ponderal index	75.8 (±0.9)	374	50.5 (±2.7)	26	-	-
Birth blood glucose (mmol)	1.5 (±0.04)	306	3.2 (±1.3)	13	1.13	0.258
Farrowing traits						
Birth order^1^	7	388	13	26	4.25	<0.001
Birth interval (mins)	18.8 (±1.3)	387	36.5 (±9.9)	26	2.38	0.017
Cumulative farrowing duration (mins)	128.6 (±4.9)	387	230.4 (±21.5)	26	0.53	0.598
Litter size	14.0 (±0.2)	388	15.0 (±0.5)	26	0.19	0.856
Sow traits						
Gestation length (days)	113.9 (±0.1)	388	113.6 (±0.2)	26	−0.86	0.389
Condition score (1–5 scale)	3.1 (±0.03)	388	2.9 (±0.1)	26	1.42	0.155
P_2_ backfat (mm)	12.9 (±0.2)	342	12.9 (±0.6)	26	−1.67	0.096

1 Birth order results presented by medians.

2 Test statistic and associate P-values for survival indicators from the GLMMs for each group of traits. See text for details.

**Table 3:** Postnatal survival indicators (means and standard errors (SE)) comparing surviving piglets with those born alive but dying pre-weaning.

Covariates	Surviving (± SE)	n	Died (± SE)	n	z-statistic^2^	P-value
Piglet traits						
Birth weight (g)	1552.3 (±17.0)	386	1237.9 (±54.4)	63	-	-
Crown-rump length (cm)	27.5 (±0.1)	374	25.5 (±0.4)	60	0.99	0.324
Body mass index	20.6 (±0.2)	374	18.7 (±0.6)	60	3.19	0.001
Ponderal index	75.8 (±0.9)	374	74.3 (±2.4)	60	-	-
Weight at 24 h (g)	1649.7 (±18.4)	388	1288.6 (±63.6)	55	-	-
%Weight change	6.6 (±0.5)	386	0.6 (±2.0)	54	−1.89	0.059
Birth temperature (°C)	37.5 (±0.1)	382	36.7 (±0.2)	60	−0.95	0.343
1 h temperature (°C)	37.7 (±0.1)	375	36.9 (±0.3)	50	3.53	<0.001
24 h temperature (°C)	38.4 (±0.1)	387	37.6 (±0.2)	41	0.52	0.600
Birth blood glucose (mmol)	1.5 (±0.04)	306	1.5 (±0.1)	42	−0.21	0.830
Blood glucose at 24 h (mmol)	4.8 (±0.1)	385	3.7 (±0.3)	40	0.13	0.899
Piglet behavioural traits						
Vitality score	3.1 (±0.04)	381	3.1 (±0.1)	62	−0.71	0.476
Latency to right (seconds)	11.3 (±0.3)	384	12.0 (±0.7)	57	−0.74	0.457
Latency to udder (mins)	12.2 (±0.8)	353	13.0 (±2.2)	53	0.41	0.680
Latency to teat (mins)	18.2 (±1.2)	340	28.1 (±6.2)	49	-	-
Latency to suckle (mins)	30.7 (±2.0)	337	41.7 (±7.6)	48	−1.38	0.167
Farrowing traits						
Birth order^1^	7	388	6.5	64	1.32	0.187
Birth interval (mins)	18.8 (±1.3)	387	16.4 (±2.8)	63	−1.41	0.159
C. farrowing duration (mins)	128.6 (±4.9)	387	119.0 (±13.8)	63	0.95	0.342
Litter size	14.0 (±0.2)	388	15.0 (±0.3)	64	−2.29	0.022
Sow traits						
Gestation length (days)	113.9 (±0.1)	388	114.0 (±0.2)	64	1.50	0.134
Condition score (1–5 scale)	3.1 (±0.03)	388	3.0 (±0.1)	64	0.75	0.452
P_2_ backfat (mm)	12.9 (±0.2)	342	12.6 (±0.5)	57	0.03	0.977

1 Birth order results presented by medians.

2 Test statistic and associate P-values for survival indicators from the GLMMs for each group of traits. See text for details.

### The differences between sexes

#### Piglet size and shape

**Figure 1:**
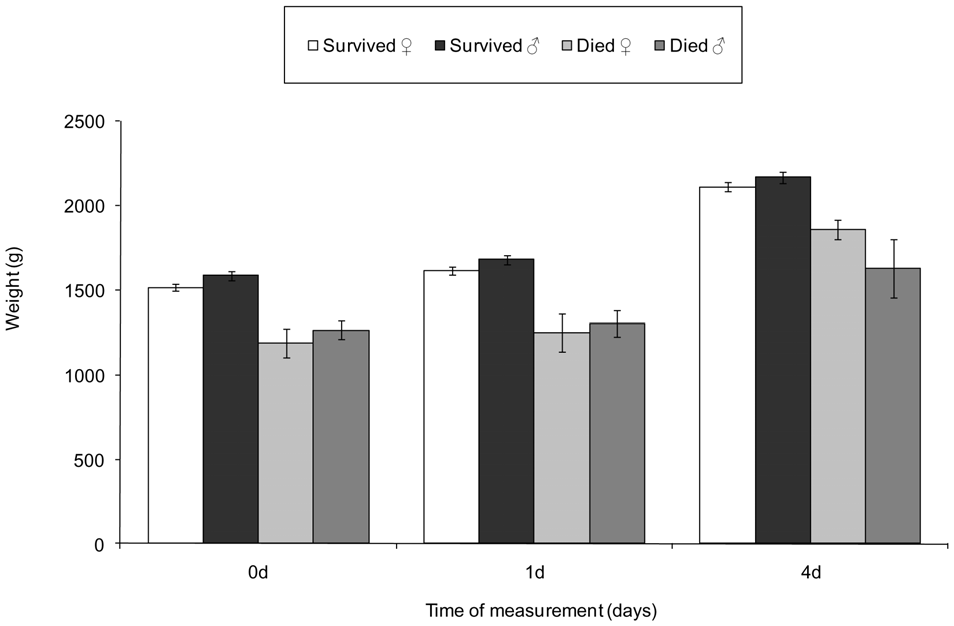
Plot of mean weights (±SE) illustrating differences between females (♀) and males (♂) that survived to weaning (Survived) and died before weaning (Died) in body weight at birth (0 d), one day (1 d) and four days (4 d) after birth. LMM analysis shows that sex and mortality significantly affect average weight across time (see text for details).

When comparing stillborn with surviving piglets, mean body mass index was significantly different between the sexes (t_364_ = 2.99, P = 0.003), with males having higher BMI on average. When comparing sexes in the postnatal model, males had significantly higher mean BMI than females regardless of survival status (Male survivors = 20.96 (±SE 0.26), died = 19.06 (±SE 0.70); Female survivors = 20.13 (±SE 0.25), died = 17.79 (±SE 0.92), t_398_ = 2.22, P = 0.027). Body weight continued to be measured over time and weights were statistically significantly different with respect to survival status (z = −5.43, P<0.0001) and sex (z = 2.11, P = 0.034), with surviving piglets being heavier than those that died and the male-bias maternal allocation evident in surviving males at birth and 24 h (Figure 1). However the differences in weight evolution between the sexes showed that, despite the initial average male-biased maternal allocation at birth, males did not sustain this difference over time; by weaning, sex allocation was only marginally male-biased (0.51 (±0.01)), and the difference in mean individual weight was not statistically significant (Males = 8288 g (±119) vs. Females = 8078 g (±116), z = 1.45, P = 0.15).

#### Thermoregulation

**Figure 2:**
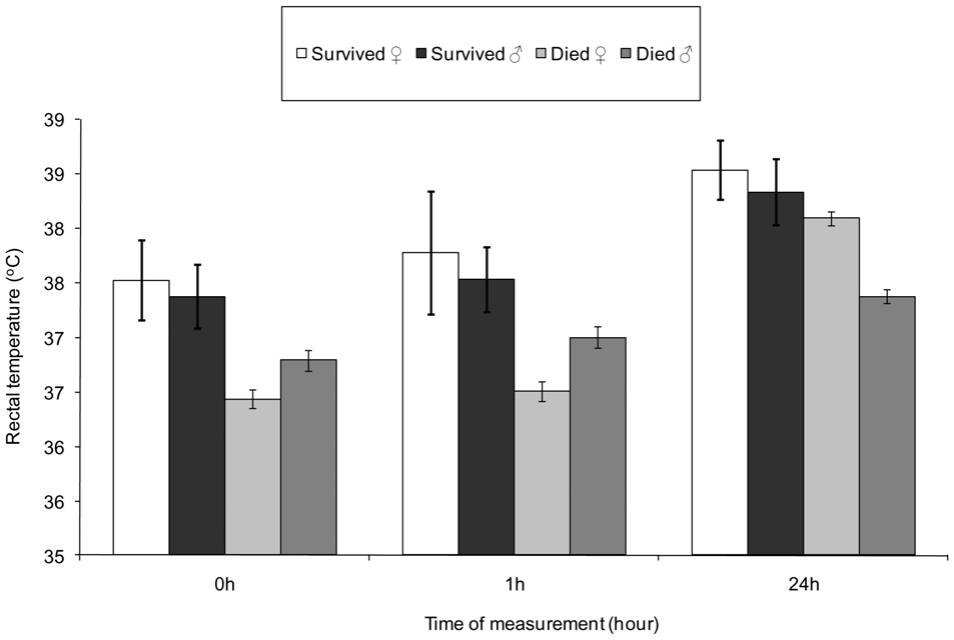
Plot of mean rectal temperatures (±SE) illustrating temperatures at birth, 1 h after birth and 24 h after birth between females (♀) and males (♂) that survived to weaning (Survived) and those that died before weaning (Died).

Figure 2 illustrates the trend for female surviving piglets to maintain higher mean rectal temperatures than male survivors. Piglet weight evolution was used as a covariate by associating birth weight with both birth temperature and 1 h temperature and 24 h weight with 24 h temperature and fitting together with sex in repeated measures LMMs to test whether the differences in mean temperature between the sexes were associated with weight. Though weight significantly influenced mean rectal temperature (z = 7.99, P<0.0001), with lower weighted individuals having lower rectal average temperatures, when rectal temperature was modelled over time, mortality and sex continued to show an influence; surviving piglets maintained significantly higher mean rectal temperatures than those that subsequently died (z = −6.82, P<0.0001) and females tended to maintain higher average rectal temperatures than males (z = −1.74, P = 0.082) (Figure 2).

### Relationship between sow condition and sex ratio

Maternal condition did not vary considerably (CS = 3.1 (±0.1) and P_2_ = 13.0 mm (±0.6)) and there was no evidence that sex ratio at birth was correlated with maternal condition at farrowing (CS: r_s_ = −0.11, P = 0.53. P_2_: r_s_ = 0.08, P = 0.67).

## Discussion

The male-biased sex allocation at birth in this study supports the theory first outlined by Clutton-Brock [21] that the relationship that exists between early development and adult size has led to selection favouring heavier birth weights in males. The males in this study received or required greater maternal resources during gestation, thus being born heavier with higher body mass index than females. However, despite evidence for initial maternal investment in males, male-biased postnatal mortality exists and reflects the greater susceptibility of this sex to causal mortality factors which can be associated with energetic demands.

The ability for the neonatal piglet to preserve homeothermy is a known critical postnatal survival indicator [32], [33], [35]. Despite a great deal of variation, males showed a trend towards lower rectal temperatures, particularly at 24 h after birth than females. The poorer thermoregulatory abilities and the significant difference between the sexes in the number of animals dying from disease-related causes further suggest male vulnerability. The most likely cause of these differences is the “decision” on which physiological processes to prioritise for investment of energy. The organism's growth, development and reproductive fitness are dependent on energetic processes and, where energy is invested, this is subject to certain trade-offs [39]. The most important trade-off regarding this particular population is that between survival and reproduction. The majority of maternal investment is directed towards (i) body size, (ii) body composition and (iii) specific physiological systems [40]. Male piglets could be directing more energy towards the first two processes, because increasing size will increase reproductive fitness in adulthood [3] and females may be directing more energy into the third process, including preserving homeothermy, immuno-competence and thus augmenting short-term survival. The former two processes of homeothermy and immunity are linked [41], [42]; chilling will predispose a piglet, not only to starvation and crushing but also to disease. The energy demands for preserving homeothermy rely primarily on carbohydrates mobilised from glycogen reserves or the ingestion of colostrum soon after birth in order to increase metabolic rate [42]. Low environmental temperature affects immunity indirectly by impeding successful acquisition of colostrum by susceptible piglets [41]. Given that males were born heavier than females, and that higher birth weight has a positive effect on thermoregulation [34], we might expect males to be more successful than females in maintaining thermoregulation. However, the effect of birth weight on cold resistance declines rapidly after birth [43] and is replaced by the importance of colostrum intake. The amount of colostrum ingested and metabolised during the first 24 h is crucial for preserving homeothermy [32] and a study of piglet weight gain over the first 24 h [44] showed that piglets maximised their weight gain in the first 2 h after birth, gaining 90 g during this period which decreased to 25 g in the third hour. Only latency to first suckle colostrum was measured in the current study with no significant differences between sexes. However measuring frequency and duration of suckling bouts during these first crucial hours might have shown a difference between the sexes that could explain poor thermoregulatory abilities in the male piglets and the inability of males to maintain significantly higher weights than females.

Metabolic demand increases with body size at around body-weight^0.75^ [45]. In order to achieve their eventual larger size males, in a sexually dimorphic species, are likely to have higher metabolic demands than females, resulting in greater nutritional requirements. There is also evidence from studies in monomorphic litter bearing species such as bank voles that sons require more maternal energy than daughters regardless of size at maturity [46], suggesting that there are sex-specific factors in addition to growth that require higher energy demands in males. Regarding larger body size in sexually dimorphic species there is clear evidence of greater nutritional requirements, reflected by studies showing that males will spend a greater amount of time feeding than females and consume more milk than females, thus putting greater nutritional strain on the mother. For example, investigations in pinnipeds revealed that male fur seal pups ingested 30% more milk than female pups [47] and male sea lion pups received more milk from their mothers than female pups, although the difference reflected the larger body size of the males [48]. In the pig, after the initial 12 h phase of continuous milk let-down, it is difficult for the male to increase its feed intake as cyclical milk let-down is limited to 10–20 second bursts [49], this has the potential to render heavier male piglets more vulnerable during this phase unless they have access to high quality teats. Perhaps males spend more time in the vicinity of the udder and massaging teats to increase their chances of having a productive teat or encouraging a greater number of let-downs (cf. the “restaurant hypothesis” [31], [50], [51]). With this comes a risk of crushing [52] as well as a trade-off between the energy of massaging the teat and the unpredictable returns from that teat. For example, a study by Klaver et al. [53] demonstrated that a piglet performing active suckling lost 0.552 g of body weight per minute, with only one fifth of that weight loss occurring during resting periods. In this study more male piglets were crushed than females, perhaps a reflection of this trade-off between measures to increase milk supply and survival.

Compromised suckling success could also affect immunity, by delaying gut closure [41]. The closure of the neonatal piglet gut typically occurs within the first 48 h of life, as transfer from passive to active immunity develops [54]. Gut closure decreases the absorption of maternal antibodies, but also limits the possibility of pathogenic agents entering the systemic circulation of the piglet [55]. Failure to suckle adequately within the first 24 h of life could slow gut closure increasing the risk of pathogenic compromise [41]. Susceptible male piglets dying from disease-related causes in this study could be those with inadequate suckling success during the critical first 24 h of life.

### Are domestic sows displaying ecological strategies for reproductive success?

The TWM postulates a sex ratio bias to increase reproductive fitness according to prevailing environmental conditions [3]. Thus investigating whether maternal condition influences sex-ratio is a key aspect of this model. Chen and Dziuk [56] showed that *in utero*, male piglets occupied more space and were heavier than females when uterine resources were adequate, however males were lighter and more likely to die when resources *in utero* were limited. In the current study the non-significant male-bias sex ratio was similar to those reported in other work with both domestic (0.52 - [57] cited in [5]) and wild pigs (0.53 [22]), and the significant male-biased sex allocation at birth reflected not only by higher birth weight but also higher body mass index suggests that conditions during gestation in this study were optimal for male fetal survival. However in the current study there was no relationship between maternal body condition at farrowing and sex ratio at birth. Sows in the study were supplied regularly with adequate food and there was very little variation in maternal body condition. Therefore data were not well suited to investigate whether sows were adopting the TWM.

It appears that the sows in this study were allocating more maternal resources to males and providing an over-supply of this more susceptible sex in anticipation of male-biased mortality during the competitive lactation phase. The process of providing an oversupply of neonates is thought to be a form of parental optimism and, in the case of swine, this over-production allows replacement offspring in the event of one or more members of the litter dying [58]–[60] and is an adaptation to unpredictable conditions during lactation, particularly regarding food supply. In the case of prenatal parental optimism the limiting resource is uterine space. As the competition for uterine space begins, males appear to have an advantage as a result of their advanced growth patterns. There is a period of developmental asynchrony in pigs, where implantation is reduced in very advanced or very retarded blastocysts. A two day window of opportunity exists, which is affected by environmental cues, where the uterus is responsive (i.e. an optimal environment exists) to signals of implantation [61]. If the environmental cues indicate optimal conditions and uterine responsiveness is synchronised with male blastocyst development, then a male-biased sex ratio would be expected. A male-biased population may be further affected by the fact that males develop quicker than females once blastocysts become embryos [61], [62]. The embryos themselves can alter the uterine environment and appear to perform prenatal “siblicide” by secreting oestrogen in the form of estradiol 17β (E2β), creating a potentially hostile uterine environment for their less developed littermates, impeding elongation and resulting in degeneration of embryos. In this prenatal environment the male piglet's advanced growth rate results in a sex ratio bias at birth, however in the postnatal period there appears to be an intrinsic, size-related effect on male mortality causing a disadvantage.

As a result of larger size and the genetic profile for a faster growth trajectory, males are more susceptible than females to shortages in resources as they have increased energy demands. This susceptibility is demonstrated in the current study by greater male mortality between birth and weaning, where the resources available to the neonates are limited by sow biology as discussed above. When competition for resources favours the larger, more dominant individuals, resulting in smaller-sized sibling deaths [63], the effects of these increased energy demands may be masked. However, where resources are sparse the effects may be more visible. The competition for functional teats at the sow's udder represents a situation where resources (i.e. colostrum and milk) could become sparse and increasing litter size will worsen this situation. For susceptible neonates sheer mass is not enough to ensure suckling success; appropriate behaviour and vigour are also key factors, the latter being demonstrated to be independent of birth weight [33] and, in a test of vigour [33] males performed significantly worse than female piglets (Baxter 2008 unpublished data). Susceptible males in the current study could not maintain their weight advantage and failure to perform adequate suckling behaviour is a likely contributing factor. Future investigations examining the suckling behaviour of piglets would provide further evidence of gender differences in behaviours vital for survival.

It is important to discuss the implications of these results in the context of modern global pig production. It is possible that because the sows in this study are living under variable extensive environments, unlike those experienced by the majority of intensively managed indoor bred animals, they are responding to environmental cues and adjusting their reproductive strategies accordingly. Studying sows under more commercially relevant indoor conditions would determine whether sex-biased maternal allocation and the oversupply of the more susceptible sex is a strategy adopted regardless of management systems. Competition for limited resources at the udder is a feature of the piglets' postnatal environment regardless of management system and one that increases with increasing litter size, suggesting that this may well be a strategy displayed universally. It is important to determine whether male-biased piglet mortality exists in the intensive, indoor systems and whether the ontogeny of this disadvantage is an intrinsic size-related effect as suggested in the current study. Reducing piglet mortality is an important goal in all pig production systems and if sex-bias mortality persists it could have implications for piglet welfare and management strategies designed to help improve piglet survival. For example cross-fostering (the transfer of piglets between native and foster litters to match teat number or even weight distribution) may be a more challenging activity for males rather than females, as transfer from the native litter to a foster litter requires appropriate behaviours to acquire and maintain a new teat. In addition if males are likely to be more immune-compromised than females, as suggested in this study, untimely removal from the native mother's colostrum and milk supply, containing valuable immunoglobulins as well as dam specific lymphocytes to facilitate cell mediated immunity [64], [65], could be more detrimental for males than females.

### Conclusions

Greater susceptibility to mortality of the male sex under challenging conditions, such as those associated with competition for functional teats at the udder, is evident from this study. Male piglets displayed poorer thermoregulatory abilities and died more from disease-related causes than females. Susceptible males showed a poor growth trajectory and it is likely that a trade-off between the necessary body size needed to maximise future fecundity and the various physiological adaptations, including thermoregulation and immuno-competence, promoting short-term survival occurred. Studying the prevalence of male-biased mortality under different management systems and its ontogeny is an important research goal with implications for strategies to improve piglet survival.
